# Pharmacotherapy for Non-Alcoholic Fatty Liver Disease: Emerging Targets and Drug Candidates

**DOI:** 10.3390/biomedicines10020274

**Published:** 2022-01-26

**Authors:** Veronika A. Prikhodko, Natalia N. Bezborodkina, Sergey V. Okovityi

**Affiliations:** 1 Department of Pharmacology and Clinical Pharmacology, Saint Petersburg State Chemical and Pharmaceutical University, 14A Prof. Popov Str., 197022 St. Petersburg, Russia; sergey.okovity@pharminnotech.com; 2 Zoological Institute, Russian Academy of Sciences, 1 Universitetskaya emb., 199034 St. Petersburg, Russia; natalia.bezborodkina@zin.ru; 3 Scientific, Clinical and Educational Center of Gastroenterology and Hepatology, Saint Petersburg State University, 7/9 Universitetskaya emb., 199034 St. Petersburg, Russia

**Keywords:** non-alcoholic fatty liver disease, non-alcoholic steatohepatitis, chronic liver disease, hepatoprotection, metabolic disorders

## Abstract

Non-alcoholic fatty liver disease (NAFLD), or metabolic (dysfunction)-associated fatty liver disease (MAFLD), is characterized by high global incidence and prevalence, a tight association with common metabolic comorbidities, and a substantial risk of progression and associated mortality. Despite the increasingly high medical and socioeconomic burden of NAFLD, the lack of approved pharmacotherapy regimens remains an unsolved issue. In this paper, we aimed to provide an update on the rapidly changing therapeutic landscape and highlight the major novel approaches to the treatment of this disease. In addition to describing the biomolecules and pathways identified as upcoming pharmacological targets for NAFLD, we reviewed the current status of drug discovery and development pipeline with a special focus on recent evidence from clinical trials.

## 1. Introduction

Non-alcoholic fatty liver disease (NAFLD) includes a range of chronic conditions characterized by excessive hepatic lipid accumulation, defined by the presence of steatosis in >5% of hepatocytes, in the absence of significant alcohol consumption or other causes of liver injury [1]. The global prevalence of NAFLD is currently estimated at 25% [2], and is projected to increase by 21% by 2030 [3]. About 20–25% of NAFLD cases are classified as non-alcoholic steatohepatitis (NASH), which has a substantially higher risk of progression to liver fibrosis, cirrhosis, and end-stage liver disease, and hepatocellular carcinoma (HCC) [3]. Considering the highly heterogeneous pathogenesis of metabolic liver diseases, the term ‘metabolic (dysfunction)-associated fatty liver disease’ (MAFLD) has recently been proposed as a broader alternative to the conventional ‘NAFLD’ [4].

Due to the increasingly high medical and socioeconomic burden of this disease as well as its strong association with obesity, metabolic and cardiovascular disorders, NAFLD pharmacotherapy has been the focus of latest research [3,5]. However, to date, no drug has been approved by neither the European Medicines Agency nor the United States Food and Drug Administration (FDA) for NAFLD [2,6].

In the present paper, we aimed to review the most recent data on major pharmacological targets in this disease, summarize the clinical evidence for novel investigational agents as well as currently marketed drugs, and highlight the latest advances in the drug development pipeline for NAFLD.

## 2. THRβ Agonists

Thyroid hormone receptor beta (THRβ) is a nuclear receptor and a transcription factor that mediates the genomic effects of thyroid hormones. THRβ1 is the predominant isoform expressed in the human liver, while other THRs are also found in the heart, brain, kidney, skeletal muscle, and other tissues. Upon activation by triiodothyronine or other agonist, THRβ displaces corepressor proteins from the deoxyribonucleic acid (DNA) and facilitates coactivator binding, thus allowing for gene transcription. THRβ can form heterodimers with other THR or nuclear receptor superfamily proteins, such as liver X receptor or peroxisome proliferator-activated receptors (PPAR) [7].

Hepatic THRβ upregulate free fatty acid (FA) uptake and oxidation, lipophagy and lipolysis, and promote mitochondrial biogenesis and respiration, leading to increased adenosine triphosphate (ATP) consumption and energy expenditure. THRβ have also been shown to induce transcriptional activation of bile acid (BA) synthesis, biliary lipid secretion, and cholesterol serum clearance, leading to a decrease in proatherogenic lipoprotein levels [8,9]. Additionally, THRβ stimulates normal hepatocyte proliferation, at the same time downregulating nuclear signaling pathways and inhibiting tumour growth and metastasis formation in HCC [10]. 

Selective THRβ agonists that are currently being developed for the treatment of NAFLD include resmetirom (MGL-3196), VK2809 (MB 07811), TERN501, ASC41, and MGL-3745. In a 36-week phase 2 randomized clinical trial (RCT) with an additional 36-week open label extension, resmetirom was safe, well tolerated, and significantly improved lipid profiles, liver steatosis (as indicated by magnetic resonance imaging/proton density fat fraction (MRI-PDFF)), liver stiffness (assessed by transient elastography), and N-terminal type III collagen pro-peptide (Pro-C3) levels in patients with biopsy-confirmed non-alcoholic steatohepatitis (NASH) [11], supporting its further evaluation in three ongoing phase 3 trials (NCT04197479, NCT04951219, NCT03900429). The lipid-lowering effect of resmetirom was accompanied by a significant improvement in alanine aminotransferase (ALT) and γ-glutamyl transpeptidase (GGT) levels as well as a reduction in NAFLD activity (NAS) and enhanced liver fibrosis (ELF) scores [11]. 

VK2809 (MB07811) is a prodrug yielding an active metabolite via oxidation by hepatic CYP3A4 enzyme. A 12-week phase 2a RCT found VK2809 to possess significant antisteatotic activity in NAFLD patients [12]. ASC41, also a prodrug, improved serum lipids and liver histology in rats [13], and subsequently demonstrated a good safety profile and substantial hypolipidemic activity in healthy volunteers [14], advancing to phase 2 (NCT05118360). A newer THRβ agonist, TERN-501, reduced serum cholesterol levels and attenuated liver steatosis and fibrosis in rodent models of hyperlipidaemia and NASH [15], and has been recently approved for phase 1 clinical trials [16,17]. MGL-3745 is being evaluated in preclinical studies, and no data regarding its therapeutic activity are available yet. Two fixed-dose combinations (FDC), ASC43 and ASC45, containing ASC41 and either a FA synthase (FASN) inhibitor (ASC40) or a farnesoid X receptor (FXR) agonist (ASC42), are being evaluated in preclinical and phase 1 clinical trials, respectively [16].

## 3. Lipogenesis Inhibitors

Acetyl-CoA carboxylase (ACC) is a multi-subunit enzymatic complex that catalyzes the irreversible carboxylation of acetyl-coenzyme A (CoA) to produce malonyl-CoA. Acetyl- and malonyl-CoA are then utilized by FASN to synthesize palmitate, which is converted to stearate by the elongation of long-chain fatty acids family member 6. Stearoyl-CoA desaturase 1 (SCD1) converts stearoyl-CoA and palmitoyl-CoA into unsaturated fatty acids, which are later esterified with glycerol by several transferases, including diglyceride acyltransferase (DGAT), to produce triacylglycerides (TAG). Two DGAT isozymes have been identified so far: DGAT1 and DGAT2, which appear to be primarily responsible for esterifying exogenous and endogenous fatty acids, respectively [18]. ACC, FASN, SCD1, and DGAT catalyze the rate-limiting steps in the de novo lipogenesis, and have therefore been considered suitable targets for the treatment of NAFLD.

However, some animal evidence suggests that inhibition of TAG synthesis may worsen hepatic inflammation and fibrosis [19], and alter intestinal barrier function, leading to diarrhoea and steatorrhoea [20]. These findings might represent a challenge for the development of lipogenesis inhibitors and potentially limit their clinical application to early stages of liver steatosis not associated with inflammation and fibrogenesis [21].

### 3.1. ACC Inhibitors

Firsocostat (GS-0976) and clesacostat (PF-05221304) are selective ACC inhibitors currently under development for combination therapy of NASH. Since ACC is indirectly inhibited by FXR [22], firsocostat has been combined with the FXR agonist (see 4.1 FXR agonists) cilofexor in order to maximize the resulting effects. In the phase 2b ATLAS trial, 20 mg/d firsocostat + 30 mg/d cilofexor provided significant reductions in NAS scores, liver steatosis, lobular inflammation and hepatocellular ballooning (HCB), and improved liver biochemistry over 48 weeks in patients with septal fibrosis due to NASH [23]. Clesacostat (2–50 mg/d) has demonstrated good efficacy in terms of reducing liver steatosis, and is being developed in combination with the DGAT2 inhibitor ervogastat to address the frequently observed elevation of serum TAG, a known effect of ACC inhibitors [24]. Two phase 2 studies are ongoing to determine the optimal doses of both agents for NASH with and without liver fibrosis (NCT04399538, NCT04321031).

### 3.2. FASN Inhibitors

Known investigational FASN inhibitors include ASC40 (TVB-2640), FT-8225, and ASC44F, a fixed-dose combination containing ASC40 and ASC42 (a FXR agonist; see *FXR agonists*). ASC40 (50–150 mg/d) provided near complete (~90%) inhibition of de novo lipogenesis and reduced liver steatosis in obese subjects with substantial NAFLD risk [25]. In the phase 2 FASCINATE-1 trial, ASC40 at lower doses (25 or 50 mg/d) improved ALT and low-density lipoprotein (LDL) cholesterol levels, attenuated liver steatosis, fibrosis, lipotoxicity, dyslipidemia, and hepatic insulin resistance in obese NASH patients [26]. A subsequent phase 2 study, FASCINATE-2, has been initiated (NCT04906421). The anti-inflammatory and antifibrotic properties of ASC40 have been linked to inhibited proinflammatory cytokine production, T-cell differentiation, and repression of collagen synthesis [26]. FT-8225 and ASC44F have entered preclinical development [16,27]; no data regarding their efficacy is available yet.

### 3.3. SCD1 Inhibitors

Aramchol (C20-FABAC) is a synthetic conjugate of arachidic and cholic acids and the first-in-class inhibitor of SCD1. In addition to inhibiting de novo lipogenesis and hepatic stellate cell (HSC)-mediated fibrogenesis [28], aramchol is capable of reducing serum cholesterol levels and promoting gallstone dissolution via stimulation of macrophage cholesterol efflux and solubilization of cholesterol [29]. In the recently completed 52-week phase 2b ARREST trial, aramchol (600 mg/d) did not meet the primary endpoint (a significant decrease in hepatic TAG), but was well tolerated and improved liver histology and ALT levels [30]. The phase 3 ARMOR RCT has been designed to evaluate the safety and efficacy of 300 mg/d aramchol in patients with biopsy-confirmed liver fibrosis due to NASH (NCT04104321).

### 3.4. DGAT Inhibitors

Ervogastat (PF-06865571), a selective DGAT2 inhibitor, significantly reduced liver fat fraction in patients with mild NAFLD [31]. Two phase 2 RCT are underway to assess the safety and efficacy of ervogastat alone and in combination with clesacostat in NASH patients with and without liver fibrosis (NCT04399538, NCT04321031). ION224 (IONIS-DGAT2_Rx_) is a ligand-conjugated chimeric antisense oligonucleotide designed to suppress the biosynthesis of DGAT2. ION224 reduced total liver fat content and several fibrosis biomarker levels in a small-scale trial in patients with type 2 diabetes mellitus (T2DM) and NAFLD [32], and is planned to be evaluated in a longer phase 2 RCT in nondiabetic subjects (NCT04932512). Newest DGAT2 inhibitors include PF-07202954, and DGAT1 inhibitors, VK1430, SNP-610, and SNP-630; the latter two molecules are reported to have additional CYP2E1-inhibiting properties [33].

### 3.5. ω-3 PUFAs

ω-3 Polyunsaturated fatty acids (ω-3 PUFA) are long-chain FA characterized by the presence of a double bond three atoms away from the terminal methyl group in their chemical structure. The three most common biologically active ω-3 PUFA include α-linolenic (ALA) acid and its metabolites eicosapentaenoic (EPA) and docosahexaenoic (DHA) acids. ω-3 PUFAs cause transcriptional repression of the key enzymes involved in hepatic glycolysis and de novo lipogenesis, such as ACC, FASN, and L-pyruvate kinase [34].

Increased ω-3 PUFA intake results in their increased incorporation into cell membrane phospholipids, corresponding to a positive shift in the ω-3/ω-6 ratio. This leads to decreased availability of the ω-6 arachidonic acid (AA), and the subsequent substrate-dependent inhibition of eicosanoid inflammatory mediator production by the leukocytes. Moreover, EPA and DHA have been found to suppress leukocyte chemotaxis and adhesion molecule expression, altogether providing a multimodal anti-inflammatory effect [35]. In addition, ω-3 PUFA are also known for their prominent antioxidant, regenerative, and antitumour properties [36].

A 6-week treatment with ω-3 PUFA (64% ALA + 21% EPA + 16% DHA) was associated with significant improvement of hepatic proteomic and plasma lipidomic markers of lipogenesis, lipotoxicity, oxidative stress, and mitochondrial respiration in patients with biopsy-confirmed NASH [37]. The increase in plasma ALA and DHA levels, and the subsequent decrease in AA levels correlated with the percentage of patients with improvements in NAS scores, lobular inflammation, and HCB. However, the overall liver histology and body weight were not significantly altered by ω-3 PUFA treatment [38].

A systematic review and a meta-analysis confirmed that DHA and EPA attenuate liver steatosis with no significant weight loss in adults [39], and a small-scale RCT reported the same effects for dietary DHA supplementation in children [40]. More recently, three meta-analyses, including up to 22 RCT and more than 1300 patients, found ω-3 PUFA to significantly decrease ALT, aspartate aminotransferase (AST), GGT levels, liver fat content and insulin resistance, having no significant effect on body weight in NAFLD [41,42,43].

Epeleuton (15-hydroxyeicosapentaenoic acid ethyl ester, DS102) is a second-generation synthetic EPA derivative. In a 16-week phase 2a RCT in obese subjects with NAFLD, epeleuton (2 g/d) improved circulating inflammatory markers, lipid profiles, and insulin resistance, but failed to reach either of the primary endpoints including reductions in ALT levels and liver stiffness [44]. Since a slight dose-dependent attenuation of liver steatosis was observed, further trials of longer duration are planned [45].

Icosabutate (NST-4016) is a structurally engineered ω-3 PUFA ether characterized to increased liver exposure due to direct absorption into the portal vein [46]. The 62-week phase 2b ICONA trial is underway to evaluate the efficacy of icosabutate (300 or 600 mg/d) in patients with biopsy-confirmed NASH (NCT04052516). Interim analysis data indicated significant dose-dependent decreases among both dosage groups in ALT, AST, GGT, and alkaline phosphatase (ALP) levels, while patients dosed with 600 mg/d icosabutate also had improvements in non-invasive fibrosis and inflammatory biomarker profiles [46].

## 4. Bile Acid Metabolism Modulators

### 4.1. FXR Agonists

Farnesoid X receptor (FXR), also known as bile acid receptor, is a nuclear receptor expressed at high levels in the liver and ileum. FXR acts as a BA sensor and governs a major negative feedback loop in the BA, glucose, cholesterol, and TAG metabolism [22]. Many of the effects of FXR are directly mediated by fibroblast growth factors (FGF) 19 and 21, downstream messengers whose functions are briefly described in Fibroblast growth factor analogues. Upon increased postprandial release of BA into the intestine, activated FXR induces the transcriptional repression of the rate-limiting enzyme cholesterol 7α-monooxygenase (CYP7A1) and several transporters involved in BA biosynthesis and liver uptake. CYP7A1 inhibition by FXR enhances the excretion of excessive cholesterol via the canalicular transporters into bile as well as directly into the intestinal lumen. FXR also upregulates the expression of bile salt export pump, multidrug resistance protein-3, and organic solute transporter α/β, which facilitate BA efflux from hepatocytes and maintain the enterohepatic circulation of bile [47]. FXR activation also favors bile acid conjugation and detoxification, and stimulates biliary phospholipid excretion. 

FXR and its downstream targets repress sterol regulatory element-binding protein 1 SREBP-1c, the major transcription factor for lipogenic pathways, thereby inhibiting FASN, ACC, and SCD1. In addition, prandial glucose can increase the intestinal FXR activity via post-translational modification, shifting the equilibrium towards glycogen deposition and reducing blood glucose levels [22]. The multifaceted nature of FXR has made it one of the most attractive novel targets for NAFLD therapy. Current FXR-activating drug candidates include the sterol derivatives obeticholic acid (OCA), EDP-305, INT-767, and INT-787, and the non-steroidal compounds MET409, tropifexor, cilofexor, vonafexor, TERN-101, ASC42, EDP-297, HPG1860, and HPG7233.

OCA (Ocaliva^®^), the first-in-class FXR agonist, is approved by the FDA for non-cirrhotic primary biliary cholangitis (PBC) and is nearing approval for liver fibrosis due to NASH [48]. In phase 2 studies, OCA increased insulin sensitivity and reduced markers of liver damage in patients with T2DM and NAFLD [49], and reduced ALT levels, improved NAS scores, prevented and partially reversed fibrogenesis in non-diabetic, pre-cirrhotic NASH patients [50]. Currently, the efficacy and safety of OCA are being evaluated in two phase 3 trials, the REGENERATE trial in NASH/fibrosis patients (NCT02548351), and the REVERSE trial in patients with compensated cirrhosis due to NASH (NCT03439254).

OCA and especially its taurine and glycine conjugates are known to actively bind Takeda G-protein receptor 5 (TGR5), a cell membrane G protein-coupled receptor (GPCR) that has been largely implicated in BA-associated pruritus development [51] and gallstone formation [52]. Hence, several newer molecules, e.g., EDP-305 and INT-787, have been structurally optimized in an attempt to avoid TGR5 engagement and potential safety concerns [53]. However, PBC trials have shown that highly selective FXR agonists retain their adverse effects, at the same time losing in overall efficacy [54,55]. EDP-305 was later repurposed for use in NASH, and, along with its close analogue EDP-297, is now intended to be reserved for future combination regimens following mixed interim results of the phase 2b ARGON-2 study [56]. Since FXR and TGR5 seem to exert additive metabolic effects, dual FXR/TGR5 agonists with balanced activity towards both targets, such as INT-767 and BAR502, have been proposed for further development for NAFLD treatment [57,58].

MET409, a structurally optimized fexaramine-derived FXR agonist with greater affinity towards intestinal rather than hepatic FXR, is characterized by improved efficacy and a differentiated adverse effect (pruritus and increased LDL-cholesterol levels) profile compared to OCA [59], and is currently being evaluated in a phase 2a RCT alone and in combination with empagliflozin (NCT04702490).

Tropifexor (LJN452), cilofexor (GS-9674), and vonafexor (EYP001) represent a group of non-steroidal small molecules with structures different from both OCA and MET409, resulting in a differential pattern of FXR-related gene expression [60] and possibly improved anticholestatic activity. The parent compound for this group, turofexorate (WAY-362450), completed a phase 1 study, but its development was discontinued thereafter [61]. Tropifexor (200, but not 140 mcg/d) reduced ALT, GGT levels, body weight, and liver fat content, and attenuated liver fibrosis in patients with biopsy-confirmed NASH in the 48-week phase 2 FLIGHT-FXR RCT [62,63]. Two additional phase 2 trials are currently underway to investigate the efficacy of tropifexor combinations with licogliflozin (NCT04065841) and LYS006 (NCT04147195) in NASH/fibrosis and NAFLD/NASH, respectively. 

Cilofexor reduced liver fat content (100 mg/d) and GGT, N-terminal type IV collagen pro-peptide, and serum primary BA levels (30 or 100 mg/d) over 24 weeks, but did not affect liver elasticity and ELF scores in NASH patients [64]. A phase 2 RCT has been initiated to evaluate the efficacy of a fixed-dosed combination of cilofexor and firsocostat, alone or in combination with semaglutide, for compensated cirrhosis due to NASH (NCT04971785). Vonafexor (100 mg/d for 12 weeks), a 2nd generation, highly selective non-steroidal FXR agonist, has recently been found to improve liver steatosis, the fibro-inflammation marker cT1 levels, and estimated glomerular filtration rate (eGFR) in NASH patients with normal or mildly decreased eGFR [65]. Nidufexor (LMB763), a non-steroidal partial FXR agonist, was being developed for NASH, liver fibrosis, and cholestatic liver disease, but appears to have been discontinued despite seemingly promising results from a phase 2 study in NASH subjects [66].

TERN-101 demonstrated a favorable safety profile and improved ALT levels and liver steatosis in the phase 2a LIFT RCT in patients with pre-cirrhotic NASH [67]. ASC42 treatment was associated with biochemical and histological improvements in animal NASH models [16], while no data are available yet for novel compounds HPG1860 and HPG7233 [68].

### 4.2. FGF Analogues

Fibroblast growth factors (FGF), including FGF19 and FGF21, are a family of hormone-like peptides with broad metabolic, transcriptional, and mitogenic activity. FGF19 is released by the ileal enterocytes into the enterohepatic circulation in response to postprandial FXR activation by BA. FGF21 is highly expressed in the liver and is released in response to PPARα, carbohydrate-response element-binding protein (ChREBP), and general control nonderepressible 2 kinase activation in the presence high serum glucose, high FFA and low amino acid levels [69]. FGF19 and FGF21 exert their physiological effects via activation of transmembrane complexes of the enzyme β-klotho (KLB) and its FGF co-receptors (FGFR) FGFR1c/2c/3c/4. FGF21 has the highest affinity for the FGFR1c/KLB complex, which is expressed in the adipose tissue and central nervous system, while FGF19 primarily targets FGFR4/KLB, which is found in hepatocytes [69]. 

Thus, FGF19 and FGF21 act as major downstream messengers in the FXR and PPARα signaling, and control the negative feedback loops to inhibit BA synthesis and lipolysis, respectively [69]. FGF19 represses CYP7A1, controls postprandial BA release into the intestinal lumen, inhibits hepatic gluconeogenesis, promotes glycogen deposition, and plays an important role in the regulation of hepatocyte proliferation and tumorigenesis. FGF21 lowers the preference for glucose intake, modulates energy expenditure, and promotes glucose and lipid uptake in the adipose tissue, which prevents ectopic lipid accumulation in liver and skeletal muscle [69].

The investigational FGF19 analogue aldafermin (NGM-282) (0.3–3 mg/d) failed to meet the primary endpoint of the 24-week phase 2b ALPINE 2/3 trial in NASH patients with stage 2 or 3 liver fibrosis, defined as a ≥1 NASH Clinical Research Network stage improvement in fibrosis with no worsening of NASH. However, the drug was well tolerated and was significantly superior to placebo in terms of NASH resolution, reduction of liver steatosis and non-invasive markers of liver injury [70]. The ALPINE 4 trial is ongoing to determine whether aldafermin could improve liver fibrosis and/or NASH in subjects with compensated cirrhosis (NCT04210245). 

Current FGF21 analogues include efruxifermin (AKR-001), BIO89-100, NN9500, BFKB8488A, MK-3655, and GLP-1-Fc-FGF21 D1. Efruxifermin is a fusion protein of human immunoglobulin G1 (IgG1) Fc domain linked to a modified human FGF21 with balanced affinity towards FGFR1c/2c/3c. Efruxifermin (28–70 mg/d) improved lipoprotein profiles and glycaemic control in T2DM patients, significantly attenuated liver steatosis in the 16-week phase 2a BALANCED study [71], and is now being evaluated in three more phase 2 RCTs (NCT05039450, NCT04767529, NCT03976401). BIO89-100 is a specifically engineered glycolpolyethylene glycol (PEG)-ylated FGF21 analogue that led to clinically meaningful reductions in liver fat content, markers of inflammation and fibrosis in a phase 1b/2a proof-of-concept study in NASH patients [72]. Recently, the phase 2b ENLIVEN trial of BIO89-100 for stage 2/3 fibrosis due to NASH has been initiated (NCT04929483), while two phase 1 open-label studies are still underway (NCT05022693, NCT04048135). No preclinical data is available yet for the investigational compound NN9500 [73].

BFKB8488A and MK-3655 are humanized bispecific anti-FGFR1c/KLB agonist monoclonal antibodies (mAB). BFKB8488A was safe and adequately tolerated, reduced liver steatosis in a dose-dependent fashion, and improved markers of cardiometabolic and liver health in obese T2DM patients with NAFLD [74]. The phase 2b BANFF trial has been initiated to explore the efficacy and safety of this compound in non-alcoholic liver fibrosis (NCT04171765). MK-3655 (NMG313), an insulin-sensitizing anti-FGFR1c/KLB agonist mAB, was effective against liver steatosis in obese and insulin-resistant NAFLD patients, and has proceeded into a phase 2b study in pre-cirrhotic NASH with or without T2DM (NCT04583423).

GLP-1-Fc-FGF21 D1 is a novel fusion protein incorporating a KLB-binding FGF21 variant and a glucagon-like peptide 1 receptor (GLP1R) agonist. In murine models of T2DM and obesity, GLP-1-Fc-FGF21 D1 improved liver function, serum and hepatic lipid profiles, and reduced body weight and NAS scores with an efficacy superior to either FGF21 or GLP1R agonists alone [75]. The PEGylated human recombinant FGF21 pegbelfermin (ARX-618) has recently demonstrated suboptimal efficacy in compensated cirrhosis due to NASH in the FALCON 2 trial, resulting in pending discontinuation and/or possible repurposing for other indications [76].

## 5. Fibrogenesis Inhibitors

### 5.1. Galectin Antagonists

Galectins, formerly known as S-type lectins, are a family of carbohydrate-binding proteins that are selective towards β-galactoside-containing glycans. To date, 15 subtypes of galectins family have been identified in humans, of which galectin-1, -3, and -9 are the most implicated in liver disease. Numerous experimental and clinical study results suggest that galectins play important and diverse roles in fibrogenesis, cellular immunity and inflammatory response, cell cycle regulation, apoptosis, regeneration, and tumorigenesis [77,78].

Galectin-1 promotes the proliferation, migration and activation of HSC, and the subsequent fibrogenesis via stimulation of transforming growth factor β (TGFβ)/platelet-derived growth factor signaling and disruption of cell adhesion. In HCC, galectin-1 promotes the epithelial–mesenchymal transition, cell adhesion, metastasis, and immunosuppression. However, elevated galectin-1 expression has also been found beneficial for liver regeneration, liver allograft survival, and recovery after hepatic ischemia-reperfusion injury [77]. 

Galectin-3 has been identified as a pivotal regulator in the progression of hepatitis, hepatic fibrosis, cirrhosis, and HCC. It was found to promote autocrine and paracrine HSC activation and phagocytosis, mediate the TGFβ-dependent fibrogenesis, and upregulate the expression of certain profibrogenic cytokines such as interleukin (IL) 33. The exact role of galectin-3 for liver cirrhosis is not as clear, but its increased expression has been linked to accelerated cirrhosis development and deterioration of liver function [77]. However, galectin-3 might be protective against adipose tissue inflammation, diabetes, and atherosclerosis progression, most probably due to and its ability to scavenge the proinflammatory and proapoptotic advanced glycation end products, the increased activation of the NF-κB signaling pathway, and the downregulation of NLRP3 inflammasome and IL1β expression in immune cells [77].

A systematic review and meta-analysis by An et al. found that serum galectin-3/9 levels correlate with the risk of liver failure and cirrhosis, and high galectin-1/3 expression is associated with poor prognosis in HCC. However, the evidence for galectin involvement in chronic liver disease remains controversial, and the impact of galectin-3 levels on NAFLD/NASH is thought to be dependent upon the stage and severity of liver damage [78]. Hence, modern galectin-targeting drug candidates are intended for use in advanced NASH complicated by liver fibrosis and/or cirrhosis.

GM-CT-01 and belapectin (GR-MD-02) are semi-synthetic polysaccharides (galactomannan and galactoarabino-rhamnogalacturonan, respectively) having high affinity towards extracellular galectin-3 and, to a lesser extent, galectin-1. Both compounds promoted resolution of portal inflammation, HCB, liver fibrosis, and cirrhosis, and reduced portal pressure in a toxin-induced liver injury model in rats [79]; however, only belapectin seems to have advanced further into clinical development. In the 52-week phase 2b NASH-CX study in subjects with NASH, liver cirrhosis, and portal hypertension, belapectin (2 mg/kg/2 weeks) did not affect fibrosis or NAFLD activity, but reduced hepatic venous pressure gradient values and prevented the development of esophageal varices in a sub-group of patients [80]. The phase 2/3 NAVIGATE trial has been initiated to further evaluate belapectin in patients with liver cirrhosis due to NASH and clinical signs of portal hypertension but without esophageal varices at baseline (NCT04365868).

GB1211, an oral small-molecule selective galectin-3 antagonist, was effective in several preclinical fibrosis models, and well tolerated in human volunteers. A phase 1/2a trial in NASH/fibrosis patients was approved but subsequently placed on hold due to an undisclosed change in the clinical development strategy for this compound (NCT04607655).

### 5.2. TLR4 Antagonists

Toll-like receptor 4 (TLR4) belongs to the family of pattern recognition receptors that activate the innate immune system by recognizing their major ligand, lipopolysaccharide (LPS). In the liver, TLR4 are expressed in both parenchymal and non-parenchymal type cells. TLR4 activation results in the activation of NF-κB, mitogen-activated protein kinase and interferon regulatory factor-mediated pathways, and the subsequent inflammatory cytokine and interferon production [81].

TLR4 stimulate adhesion molecule expression and chemokine secretion by the HSC, which induces Kupffer cell migration and the recruitment of extrahepatic monocytes into the liver. TLR4 also downregulate HSC expression of the Bambi protein, an endogenous TGFβ receptor inhibitor, thus promoting profibrogenic TGFβ signaling. Additionally, TLR4 can inhibit miR-29 expression and increase fibronectin production in the HSC, further enhancing HSC activation and migration [82].

JKB-122 treatment (5 or 35 mg/d for 12 weeks) was well tolerated and significantly improved ALT and AST levels, liver steatosis, and serum lipid profiles in a phase 2 study in patients with NAFLD [83]. Another phase 2 RCT is planned to investigate whether JKB-122 could ameliorate liver fibrosis due to NASH (NCT04255069). Recently, eritoran, a synthetic bacterial lipid analogue, was found to significantly reduce ALT levels, lobular inflammation, intrahepatic neutrophil infiltration, and liver fibrosis, but not liver steatosis, in murine models of acute and chronic liver injury [81].

### 5.3. LOXL2 Inhibitors

Lysyl oxidase-like protein (LOXL) 2 is a histone modifier amine oxidase that has been identified as the primary enzyme facilitating covalent crosslinking of collagen and elastin fibers, thereby promoting collagen network formation and progression of liver fibrosis. LOXL2 inhibition following the onset of fibrosis has been demonstrated to augment and accelerate collagen degradation in rodent models [84]. Only a few studies have concerned the role of LOXL3, a less common isozyme of LOXL, in fibrotic diseases, and its value as a therapeutic target is somewhat controversial [85].

Simtuzumab (GS-6624), a humanized antagonist mAB, was one of the first LOXL2-targeting drug candidates, that was discontinued after showing a lack of efficacy regarding liver fibrosis and/or portal hypertension in two phase 2b trials in patients with bridging fibrosis or compensated cirrhosis associated with NASH [86]. 

Novel LOXL2 antagonists are represented by orally available small molecules. PXS-5153A, a dual LOXL2/LOXL3 inhibitor, reduced disease severity and improved liver function by diminishing collagen content and collagen crosslinks in two rodent models of liver fibrosis [87]. Its cognate PXS-5382A, selective towards LOXL2, showed a satisfactory pharmacokinetic profile in healthy volunteers (NCT04183517), and is expected to advance into phase 2 trials. Another LOXL2 inhibitor, GB2064, was initially researched for myelofibrosis, but appears to have had its potential indication list expanded to include other fibrotic diseases [88].

### 5.4. ATX Inhibitors

Autotaxin (ATX) is a glycoprotein enzyme that converts membrane-derived lysophospholipids into lysophosphatidic acid (LPA). LPA, in turn, acts as a multimodal signaling molecule and causes cytoskeleton remodelling, alters cell proliferation and migration, and promotes fibrogenesis as well as inflammatory reactions [89]. Non-competitive inhibition of autotaxin/LPA signaling by the indole derivative PAT-505 resulted in a significant attenuation of liver fibrosis in mouse models of NASH [90]. In addition, ATX induction has been implicated in BA-mediated pruritus development, suggesting potentially favorable safety profiles of ATX inhibitors. Two small-molecule ATX inhibitors, TJC0265 and TJC0316, have been identified as lead compounds and are currently undergoing optimization and preliminary in vivo testing [91].

## 6. Glucose Metabolism Modulators

### 6.1. PPAR Agonists

PPAR are a family of nuclear receptors that function as transcription factors and play a regulatory role in glucose homeostasis, lipid metabolism, inflammatory response, cell development and differentiation [92]. Up to now, three PPAR subtypes have been identified: (1) PPARα, expressed in the liver, adipose tissue, skeletal muscle, heart, and kidney; (2) PPARγ, expressed in the adipose tissue, colon, macrophages, pancreas, skeletal muscle, etc.; and (3) PPARδ(β), found ubiquitously. Upon ligand binding, PPAR induce the release of corepressors and recruitment of coactivators, allowing for gene transcription. Moreover, PPAR can regulate the mitogen-activated protein kinase pathways, and inhibit inflammatory reactions via transrepression of several proinflammatory transcription factors such as NF-κB [92].

Hepatic PPARα stimulate mitochondrial FA uptake, β-oxidation, ATP production, and ketogenesis. They upregulate glucose-sensing transcription factors ChREBP and sterol regulatory element-binding transcription factor 1, and promote FGF21 expression, which increases tissue insulin sensitivity and maintains glucose homeostasis. Recent experimental studies strongly suggest that decreased hepatic PPARα expression positively correlates with insulin resistance and NASH severity, while NASH resolution is associated with an upregulation of PPARα as well as its target genes [92]. However, the evidence for the use of selective PPARα agonists in NAFLD is limited. 

Fenofibrate (200 mg/d) induced complete resolution of biochemical and ultrasonographic evidence of NAFLD in almost half of the patients in an open-label RCT [93], but had minimal efficacy regarding liver histology [94]. Gemfibrozil (600 mg/d for 4 weeks) reduced liver enzyme levels but also did not produce any meaningful changes in liver morphology [95], while clofibrate (2 g/d) failed to show any beneficial effect in NASH patients [96]. Pemafibrate (0.2 mg/d) improved biochemical markers of liver damage and steatohepatitis according to non-invasive measures in NAFLD subjects [97], and bezafibrate reduced liver steatosis in obese mice with metabolic syndrome [98].

PPARγ are critical positive regulators of adipocyte differentiation and lipogenesis, insulin sensitivity, and glucose uptake by skeletal muscle. Additionally, PPARγ may reduce inflammation via inhibition of macrophage activation and tumour necrosis factor α production [92]. Latest EASL-EASD-EASO [1] and AASLD [99] clinical practice guidelines support the use of pioglitazone, a selective PPARγ agonist, in progressive and/or high-risk, biopsy-proven NASH, due to its efficacy regarding liver histology in NASH patients with or without T2DM. More recently, lobeglitazone has been demonstrated to reduce intrahepatic fat content and biochemical markers of liver damage in T2DM patients with NAFLD [100]. An experimental study found nifedipine, an L-type calcium channel blocker, to exert protective action against diet-induced NASH in rats, most probably due PPARγ activation [101].

PPARδ is the less studied PPAR isotype despite its ubiquitous expression. Experimental studies have reported PPARδ to reduce very low-density lipoprotein cholesterol levels, inhibit adipocyte growth and lipid uptake, prevent the formation of reactive oxygen species, and modulate Kupffer cell activation [92]. A recent study found a correlation between severe, but not moderate, hepatic steatosis and decreased hepatic PPARδ expression [102]. 

Seladelpar (MBX-8025) is the only selective PPARδ agonist currently in the pipeline for the treatment of NAFLD. The interim analysis results of a 52-week phase 2b RCT of seladelpar (10–50 mg/d) found it to reduce ALT, GGT, and ALP levels, but only minimally influence liver steatosis at 12 weeks of treatment [103]. Further trials have so far focused more on the anticholestatic properties of seladelpar, of potential value in primary biliary cholangitis (NCT03301506).

Saroglitazar (Lipaglyn^®^), a dual PPARα/γ agonist, is approved in India for use in T2DM and precirrhotic NASH. In several RCT in NAFLD patients with and without T2DM, saroglitazar (4 mg/d) was shown to significantly improve liver biochemistry as well as hepatic steatosis by non-invasive measures over 16–24 weeks of treatment [104,105]. The group of experimental triple PPARα/γ/δ agonists is now represented by lanifibranor (IVA337) alone, after its predecessor elafibranor (GFT-505) was discontinued due to lack of efficacy in NAFLD in the phase 3 RESOLVE-IT trial (NCT02704403). Lanifibranor was well tolerated and reduced liver enzyme levels and markers of fibrosis in patients with precirrhotic, highly active NASH in the recently completed phase 2b study. The percentage of patients with meaningful improvements in steatosis, activity, and fibrosis scores was significantly higher in the lanifibranor-treated arms, indicating possible improvements in hepatitis activity as well as HCB [106]. Two more trials to evaluate the efficacy of lanifibranor in concomitant NAFLD and T2DM and in advanced fibrosis due to NASH are ongoing (NCT03459079, NCT04849728).

### 6.2. MPC Inhibitors

In addition to their primary mechanism of action, pioglitazone and other thiazolidinediones are known to interact with the mitochondrial pyruvate carrier (MPC) to suppress pyruvate transport into the mitochondrial matrix. Since this direct, non-genomic effect is considered essential for the inhibition of hepatic gluconeogenesis by PPARγ ligands, novel MPC-inhibiting thiazolidinedione derivatives with minimal affinity towards PPARγ have been synthesized. 

Azemiglitazone (MSDK-0602K) reduced liver enzyme levels in NASH/fibrosis patients regardless of T2DM presence [107], and was characterized by a markedly improved safety profile [108]. However, the drug did not demonstrate significant effects on any of the histological endpoints [107]. A phase 3 trial to evaluate azemiglitazone in diabetic or prediabetic patients with NAFLD/NASH has been initiated (NCT03970031). An alternative approach to minimizing the side effects of PPARγ agonists is represented by PXL065, the deuterium-stabilized R-isomer of pioglitazone [109], which is currently being assessed in a phase 2 RCT (NCT04321343).

### 6.3. Incretin Mimetics

Incretins are a small family of intestinal L-cell-derived peptide hormones that includes glucagon-like peptide 1 (GLP1), glucose-dependent insulinotropic polypeptide (GIP), and oxyntomodulin. The incretin axis mediates the physiological response to hyperglycaemia and couples glucose intake with pancreatic secretion [110]. GLP1 receptors (GLP1R) are expressed in the β-cells, hepatocytes, white adipose tissue, brain, and skeletal muscle [111]. GLP1R activation induces insulin secretion, decreases insulin resistance, inhibits glucagon release and lipogenesis, and suppresses appetite and gastrointestinal motility. Additionally, hepatic GLP1R stimulate FA β-oxidation, inhibit profibrogenic signaling pathways, and exert a mild anti-inflammatory effect by indirectly reducing CRP, proinflammatory cytokine, and chemokine production [112]. 

At the moment, semaglutide is the only GLP1R agonist being developed for the treatment of NASH in nondiabetic subjects. In the recently completed 72-week phase 2 trial, semaglutide treatment (0.4 mg/d) led to NASH resolution with no worsening of fibrosis in 59% vs. 17% in the placebo group, which is considered the highest response rate that a drug has ever achieved in a NASH trial up to now [113,114]. Currently, semaglutide is being evaluated in several phase 2 trials as monotherapy (NCT03884075, NCT04216589) as well as in combinations with empagliflozin (NCT04639414), and cilofexor/firsocostat (NCT04971785). A 5-year-long phase 3 study designed to include 1200 patients with precirrhotic NASH has been initiated (NCT04822181).

Exenatide, lixisenatide, liraglutide, and dulaglutide have all demonstrated significant antisteatotic activity, and (with the exception of lixisenatide) improved intrahepatic cholestasis in T2DM/NAFLD patients. Amelioration of cytolysis markers was reported for exenatide [115,116] and dulaglutide [117,118], and lixisenatide was effective against liver fibrosis and inflammation [119]. Liraglutide reduced hepatitis activity and liver fibrosis as well as attenuated HCB in NASH patients regardless of the presence of T2DM, as determined by the phase 2 LEAN study [120]. A recent meta-analysis by Ghosal et al., including 8 RCT and over 600 patients, found that GLP1R agonists in general improve liver function and histology by improving glycaemia, reducing body weight and hepatic fat content, which in turn might be beneficial for hepatic inflammation in NAFLD concomitant with T2DM [121].

While both GIP and GLP1 are potent insulin secretagogues, GIP has a more robust, dose-dependent secretory pattern, and appears to make a greater contribution than GLP1 to prandial insulin secretion in healthy subjects, while in T2M its activity is depleted. In addition, GIP, but not GLP1, stimulates glucagon secretion by the α-cells at low glycaemia under physiological conditions, and in a glucose-independent fashion in T2M [110]. GIP receptors (GIPR) upregulate lipogenesis, FA esterification and TAG accumulation in adipocytes, and inhibits prandial lipid absorption. Supraphysiological levels of GIP have been associated with increased systemic inflammatory response, and are considered a risk factor for the development of NASH [122].

Tirzepatide (LY3298176) is a synthetic injectable dual GLP1/GIP peptide agonist currently researched for NAFLD treatment. In T2DM subjects with NASH, tirzepatide (1–15 mg/week for 26 weeks) effectively reduced ALT, AST, cytokeratin 18, Pro-C3, and increased adiponectin levels [123]. Additionally, tirzepatide treatment led to greater improvements in liver fat content compared to titrated insulin degludec in T2DM, according to the phase 3 SURPASS-3 RCT results [124]. A phase 2 study, designated SYNERGY-NASH, has been initiated to evaluate the efficacy of tirzepatide in nondiabetic subjects with NASH (NCT04166773).

Oxyntomodulin shares sequence similarity with both and GLP1 and glucagon, and activates GLP1R and glucagon receptors (GCGR) under physiological conditions. Simultaneous GLP1R activation prevents hyperglycaemic response characteristic of glucagon, at the same time potentiating its catabolic effects and greatly intensifying hepatic glycolysis, glycogenolysis, and lipolysis [125]. Weight reduction, anorexigenic and hypoglycaemic effects have been linked to GLP1 activation, while GCGR activation is thought to contribute primarily to hepatic steatosis attenuation and improved mitochondrial respiration. The clinical utility of oxyntomodulin itself is limited by a short circulatory half-life due to rapid renal clearance and degradation by dipeptidyl peptidase 4 (DPP4) [126].

In contrast to the native hormone, synthetic oxyntomodulin mimetics are resistant to proteolytic cleavage and have prolonged pharmacological action. Cotadutide (MEDI0382) (100–300 μg/d) caused substantial improvements in liver enzyme levels and markers of liver fibrosis in concomitant obesity, T2DM, and NASH in a phase 2b study that included over 800 subjects [127]. Efinopegdutide (HM12525A, MK-6024), a PEGylated long-acting peptide agent, has demonstrated promising antihyperlipidaemic, antisteatotic, and anti-inflammatory activity in mice and hamsters [128], and is going to be evaluated in a phase 2 RCT in NASH with semaglutide as an active comparator (NCT04944992). Other dual GLP1/GCCR agonists intended for use in NAFLD include pemvidutide (ALT-801) (NCT05006885), danuglipron (PF-06882961) (in combination with ervogastat) [129], BI 456906 (NCT04771273), and HM14320 (a glucagon-containing combination) [130]. Finally, a novel triple GLP1R/GCGR/GIPR agonist, HM15211, induced significant reductions in liver steatosis, fibrosis, and inflammation in mice [131]; a phase 2 clinical trial is ongoing (NCT04505436).

GLP2, usually not considered an incretin, is prevalent in the gastrointestinal tract, where it promotes lipid absorption, regulates intestinal motility, mucosal morphology, function and integrity of the intestine [132]. Teduglutide, a selective GLP2R agonist, reduced liver steatosis and disease activity scores in rats, possibly by restoring normal intestinal permeability [133].

DPP4 inhibitors represent a group of indirect incretin mimetics as they prevent the proteolytic cleavage of GLP1, GIP, and oxyntomodulin. To the best of our knowledge, no DPP4 inhibitors are yet in the global pipeline for liver disease. However, a number of small-scale clinical trials have evaluated their potential efficacy in NAFLD in the presence or absence of concomitant T2DM. Among this group, only sitagliptin (100 mg/d) was found effective against hepatic steatosis and HCB irrespective of T2DM in a 1-year open-label RCT [134]. Vildagliptin (100 mg/d) [135], saxagliptin (5 mg/d) [136], omarigliptin (25 mg/week) [137], and teneligliptin (20 mg/d) [138] improved liver function and some non-invasive markers of NAFLD, and alogliptin (25 mg/d) was only moderately effective against NASH over 12 months of treatment in T2DM/NAFLD patients [139]. A recent meta-analysis by dos Santos et al. found the existing evidence for DPP4 inhibitors in NAFLD to be of poor quality and altogether not supportive of their clinical effectiveness [140]. Evogliptin [141], anagliptin [142,143], trelagliptin [144], gemigliptin [145], and linagliptin [146] have demonstrated beneficial effects in experimental rodent models, but their clinical value remains to be explored in future trials.

### 6.4. SGLT Inhibitors

Sodium/glucose cotransporter (SGLT) 2 inhibitors are a relatively novel class of oral antidiabetic agents that increase urinary glucose excretion by inhibiting glucose reabsorption by SGLT2 in the proximal tubules. Several trials have demonstrated the improvement of cardiovascular and renal outcomes by treatment with compounds of this class, namely, empagliflozin, canagliflozin, and dapagliflozin [147]. SGLT2 inhibitors are known for their multiple metabolic effects that are notably relevant to NAFLD pathophysiology, including the general shift towards increased ketogenesis, gluconeogenesis, glycogenolysis, and FA β-oxidation. They inhibit leptin production by adipocytes, leading to decreased food intake, increase adiponectin levels, provide mild insulin sensitization, suppress HSC activation and fibrogenesis. Additionally, SGLT2 inhibitors may indirectly suppress sympathetic innervation and increase the vagal tone, thereby preventing the activation of Kupffer cells and the associated inflammatory processes [148,149].

Recent evidence mostly supports the efficacy of the majority of SGLT2 inhibitors for improving liver dysfunction, steatosis and fibrosis in NAFLD concomitant with T2DM. Among this group, only dapagliflozin, empagliflozin, and canagliflozin treatment was associated with beneficial effects in nondiabetic NAFLD patients. Dapagliflozin (10 mg) significantly reduced ALT, AST, and GGT levels, according to a retrospective study [150], while empagliflozin (10 mg/d) also attenuated liver steatosis and liver stiffness, indicative of potential antifibrotic activity, in a small-scale RCT [151]. The phase 3 DEAN trial to evaluate dapagliflozin in biopsy-confirmed NASH patients has been initiated (NCT03723252). Canagliflozin (100 mg/d) improved liver enzyme levels and FIB-4 index values in an open-label, uncontrolled pilot study [152]. Additionally, empagliflozin had a beneficial effect on cognitive functions and reduced anxiety in an experimental NAFLD model [153].

Ipragliflozin (50 mg/d for 72 weeks) ameliorated liver fibrosis and enhanced NASH resolution [154], remogliflozin etabonate (50–1000 mg/d for 12 weeks) reduced FIB-4 and NAFLD-fibrosis scores [155], and ertugliflozin (5 or 15 mg/d for 52 weeks) reduced liver transaminase levels [156] in TD2M patients with different stages of NASH. Several pilot studies in T2DM/NAFLD subjects confirmed the antisteatotic properties of luseogliflozin and tofogliflozin [157,158,159].

The SGLT1 subtype plays a relatively smaller (10–20% and up to 40% when SGLT2 are blocked) role in the renal glucose reabsorption, but is more abundant in the small intestine along with the heart and lungs. Intestinal SGLT1 (iSGLT1) inhibition leads to substantially reduced glucose and galactose absorption from the intestinal lumen, and increased incretin (GLP1, peptide YY) release by enteroendocrine cells [160]. Currently, licogliflozin (LIK066) is the only SGLT1/2 inhibitor being evaluated in NASH independent of T2DM presence, alone and in combination with the FXR agonist tropifexor, in the ongoing phase 2 ELIVATE study (NCT04065841). Previously, licogliflozin (150 mg/d) reduced ALT, ALT, GGT levels and liver fat content over 12 weeks compared to placebo [161]. A novel compound, SGL5213, has been identified as a selective iSGLT1 inhibitor, and has demonstrated insulin-sensitizing, anti-inflammatory, and antifibrotic activity in a murine model of NAFLD [162].

### 6.5. α-Glucosidase Inhibitors

α-Glucosidase is a carbohydrate hydrolase located in the brush border of the small intestine that catalyzes the breakdown of dietary starch and disaccharides to yield glucose. α-Glucosidase inhibitors slow down carbohydrate digestion and absorption, thereby reducing postprandial hyperglycaemia. However, they are characterized by only modest overall antidiabetic activity, and are not too often used in clinical practice [163]. Despite some scientific interest concerning the use of this class of drugs for the treatment of liver diseases, data regarding their efficacy for NAFLD remain scarce. Acarbose (100 mg/d) improved AST, ALT levels and lipid profiles, albeit to a lesser extent than ezetimibe, in a 10-week small-scale RCT in non-diabetic NASH patients [164]. Miglitol treatment (150 mg/d for 12 months) was associated with significant improvements in steatosis, lobular and portal inflammation, and NAS scores, while fibrosis and hepatocyte ballooning remained unchanged [165]. Finally, voglibose prevented hepatic steatosis in obese rats, but was slightly inferior to empagliflozin [166].

**Figure 1:**
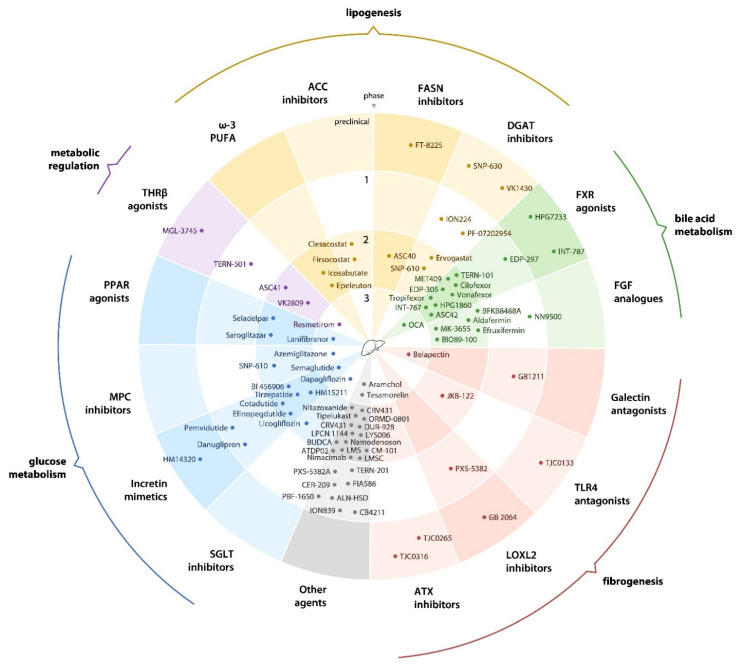
An overview of the current drug development pipeline for non-alcoholic fatty liver disease. FASN, fatty acid synthase; DGAT, diglyceride acyltransferase; FXR, farnesoid X receptor; FGF, fibroblast growth factor; TLR4, toll-like receptor 4; LOXL2, lysyl oxidase-like protein 2; ATX, autotaxin; SGLT, sodium/glucose contransporter; MPC, mitochondrial pyruvate carrier; PPAR, peroxisome proliferator-activated receptor; THRβ, thyroid hormone receptor β; PUFA, polyunsaturated fatty acid; ACC, acetyl-CoA carboxylase; BUDCA, berberine ursodeoxycholate; LMS, leucine-metformin-sildenafil; LMSC, liver-derived mesenchymal stromal cells.

An overview of the current drug development pipeline for NAFLD is given in Figure 1.

## 7. Other Agents

### 7.1. Probiotics

According to recent evidence, NAFLD pathogenesis is tightly associated with intestinal bacterial overgrowth and an upset balance between Bacteroidetes and Firmicutes species, leading to alterations in the gut-derived metabolite and endotoxin production [167]. A number of small-scale RCT in paediatric and adult NAFLD patients have demonstrated the beneficial effects of probiotics containing various combinations of *Bifidobacterium* spp. [168], *Lactobacillus* spp. [169,170], *Streptococcus thermophilus* [171], *Pediococcus pentosaceus* [172], *Lactococcus* spp., *Propionibacterium* spp., and *Acetobacter* spp. [173] Two meta-analyses of 134 and 535 NAFLD patients from 4 and 9 RCTs, respectively, have confirmed that probiotics can improve insulin sensitivity, ameliorate dyslipidaemia and systemic inflammation, reduce intrahepatic fat content, and rescue impaired liver function, overall improving the clinical outcomes in NAFLD [174,175]. Additionally, probiotics based on *Saccharomyces boulardii* [176] and *Clostridium butyricum* [177] have demonstrated antisteatotic, anti-inflammatory, and antifibrotic properties in animal models of NAFLD. Clinical evidence for probiotic use in NAFLD is summarized in Table 1.

### 7.2. Mesenchymal Stromal Cells

Lately, cell-based therapy has emerged as a feasible alternative for the treatment of different stages of NAFLD. In particular, experimental evidence supports the use of bone marrow- [187], umbilical cord- [188], and compact bone-derived mesenchymal stromal cells [189] as well as hepatocytes derived by differentiating induced pluripotent stem cells [190]. The crosstalk between hepatic stem cells and their possible therapeutic application for NAFLD are discussed in detail in a recent review by Overi et al. [191].

HepaStem^®^ is a first-in-class allogeneic stem cell therapy product containing human adult liver-derived progenitor cells with potential indications including cirrhotic and precirrhotic NASH as well as acute-on-chronic liver failure (ACLF). HepaStem^®^ cells, obtained from healthy donors, are expected to modulate the inflammatory response and inhibit HSC activation, thereby reducing liver fibrosis. A small-scale phase 2a RCT found HepaStem to be safe and well tolerated, and indicated potential efficacy for ACLF and/or decompensated liver cirrhosis [192].

### 7.3. Fraudulent Fatty Acids

Fraudulent, or abnormal fatty acids, represented by bempedoic acid (ETC-1002) and gemcabene (CI-1027), are molecules with structures similar to those of oleic or linolenic acid that regulate metabolic pathways in the liver, resulting in enhanced FA catabolism. After conversion into its active form, bempedoic acid acts as false substrate and inhibits hepatic adenosine triphosphate citrate (pro-S)-lyase, an enzyme upstream of 3-hydroxy-3-methylglutaryl-CoA reductase in the cholesterol synthesis pathway. This links fraudulent fatty acids to statins, whose possible beneficial effects for NAFLD are reviewed elsewhere [193].

Bempedoic acid is approved in the USA and EU as monotherapy and as a fixed-dose combination with ezetimibe for the treatment of hypercholesterolaemia [194]. In a high-fat diet-induced murine model of NASH, it caused significant reductions in ALT and AST levels, hepatic TAG accumulation, proinflammatory and profibrotic gene expression, resulting in improved NAFLD activity and liver fibrosis by histological analysis [195].

Gemcabene (PD-72953), a structurally optimized derivative of bempedoic acid, forms a CoA conjugate that inhibits ACC, and reduces apolipoprotein C-III expression [194]. In a mouse model of NASH/HCC, it diminished micro- and macrovesicular liver steatosis, HCB, inflammatory infiltration, and fibrosis, which corresponded to downregulated proinflammatory, lipogenesis, and profibrogenic marker expression [194]. Gemcabene was being developed for the treatment of paediatric NAFLD, but was discontinued and repurposed for another indication after a lack of efficacy was demonstrated in a phase 2a proof-of-concept study (NCT03436420).

### 7.4. Tesamorelin

Tesamorelin (TH9507) is a growth hormone (GH) releasing hormone analogue that is thought to stimulate lipolysis via increasing endogenous GH levels while maintaining feedback inhibition and limiting toxicity compared to native GH. Tesamorelin reduced liver fat content and visceral fat in a preliminary study in antiretroviral-treated patients with human immunodeficiency virus (HIV)-associated lipodystrophy [196]. A phase 2 trial to evaluate the effects of tesamorelin on liver steatosis and cardiovascular risk in obese NASH patients is recruiting (NCT03375788), and a phase 3 study in the general population with NAFLD including a HIV cohort has been planned [197].

### 7.5. Berberine Ursodeoxycholate

Berberine ursodeoxycholate (BUDCA, HTD1801) is an ionic salt of the isoquinoline alkaloid berberine and ursodeoxycholic acid (UDCA). According to a meta-analysis by Wei et al., berberine can significantly improve liver function, lipid profiles, and glycaemic control in patients with NAFLD [198] due to adenosine monophosphate-activated protein kinase (AMPK) activation, stimulation of glycolysis, and, possibly, inhibition of α-glucosidase [199]. UDCA, in turn, is a bile acid long used for the treatment of NASH and chronic cholestatic diseases, whose hepatoprotective effects are confirmed by several systematic reviews and meta-analyses [200,201]. In a phase 2 proof-of-concept RCT in T2DM patients with presumed NASH, BUDCA reduced liver enzyme levels and liver steatosis by MRI-PDFF [202].

### 7.6. Miricorilant

Miricorilant (CORT 118335) is an investigational glucocorticoid receptor agonist/antagonist and a mineralocorticoid receptor antagonist currently in development for NASH and antipsychotic-induced weight gain. Results of a phase 2a study in NASH patients demonstrated that miricorilant (600 mg/d) effectively ameliorated liver steatosis by radiological measures. However, miricorilant treatment was associated by transient yet significant increases in serum transaminases [203], and the trial was subsequently put on hold due to safety concerns (NCT03823703).

### 7.7. Nitazoxanide

Nitazoxanide (Alinia^®^) is an FDA-approved broad-spectrum thiazolide antiprotozoal and antiparasitic agent, lately reported to be a potent AMPK activator and inhibitor of HSC activation. In experimental studies in mice, nitazoxanide (100 mg/kg/d) attenuated dyslipidaemia, liver steatosis [204], fibrosis, inflammation, and HCB, demonstrating synergistic effects with the pan-PPAR agonist elafibranor [205]. Moreover, the anti-anaerobic activity of nitazoxanide may determine its use in preventing the recurrence of hepatic encephalopathy as a viable alternative to rifaximin (NCT04161053) [206].

### 7.8. Pirfenidone

Pirfenidone (Esbriet^®^) is a pyridine derivative with antifibrotic, anti-inflammatory, and antioxidant properties, the precise mechanisms of which are still unclear. In the liver, pirfenidone may decrease fibronectin, TGFβ, collagen production and attenuate fibrogenesis, hepatocyte necrosis, and necroinflammation [207,208]. In the phase 2 PROMETEO study, pirfenidone (1200 mg/d) markedly reduced transaminase levels and advanced liver fibrosis of predominantly nonalcoholic aetiology [209].

### 7.9. Miscellaneous

Other investigational drugs with potential therapeutic value in NAFLD include antileukotriene agents [210], GPCR modulators [211], anti-IL mABs [212], IL22 axis modulators [213], purinergic receptor agonists [214], antioxidants [215], antisense oligonucleotides [216], multitarget epigenetic regulators [217], and many more. A comprehensive list of drug candidates and experimental agents with evidence of hepatoprotective activity in NAFLD is given in Table 2.

## 8. Future Directions

Other biomolecules and pathways most recently identified as potential therapeutic targets in NAFLD have been highlighted in many comprehensive review articles [246,247,248,249]. Of those, the sirtuins, a family of highly conserved histone and protein deacetylases, can be considered of special interest regarding future therapeutic concepts for NAFLD. Sirtuins act as NAD^+^-sensing signaling proteins to facilitate stress response [250], maintain homeostasis during acute and chronic inflammatory response [251], and partake in the regulation of energy metabolism, redox balance, cell cycle, and suppression of tumorigenesis [252].

Sirtuin 1 (SIRT1), the SIRT1/NF-κB axis, SIRT3, and SIRT4 play a major role in regulating hepatic lipid metabolism, controlling oxidative stress, and mediating chronic inflammation in NAFLD and alcoholic fatty liver disease [253,254,255]. Accordingly, SIRT1 activation by the polyphenol resveratrol and several small molecules have been shown to provide protection against NAFLD and T2DM in rodent models [253,256], and may be beneficial in human metabolic disease [257]. SIRT2, SIRT3, and SIRT4 upregulation induced by an investigational molecule also prevented the progression of hepatic steatosis and fibrosis in obese rats [258]. While still at early research and development stages, selective sirtuin activators and inhibitors are considered a promising group of drug candidate molecules with primarily anti-inflammatory mode of action [251,254,257].

## 9. Conclusions

Being the most prevalent cause of chronic liver disease worldwide, NAFLD is at the same time one of the greatest areas of unmet medical need in terms of availability and adequacy of pharmacotherapeutic options. The modern pipeline for NAFLD includes a plethora of drug candidates with diverse and innovative mechanisms of action, although reported evidence on their clinical effectiveness has so far been limited. Upcoming treatment approaches to different stages of NAFLD include the modulation of nuclear receptor activity in order to maintain lipid and glucose homeostasis, stimulation of bile acid metabolism, and direct inhibition of fibrogenesis, along with a few less explored but highly promising options.

Despite several investigational agents being seemingly close to regulatory approval as monotherapies, novel therapeutic strategies for NAFLD will likely involve the use of multitarget drugs or rational drug combinations. Given the exceptionally complex pathophysiology and the multifaceted nature of this disease, NAFLD pharmacotherapy can be expected to remain a priority for biomedical research in the nearest future.

## Figures and Tables

**Table 1:** Probiotic formulations with evidence of hepatoprotective activity in non-alcoholic fatty liver disease.

Composition	Liver-Related Effects						
	Cytolysis	Steatosis	HCB	Inflammation	Fibrosis	Cholestasis	References
*Bifidobacterium longum*	+	+	±	±			[168]
*Lactobacillus acidophilus*	+						[178]
*L. acidophilus*, *B. lactis*	+						[170]
*L. rhamnosus*	+						[169]
*L. plantarum* *	+						[179]
*L. paracasei* *	+	+					[180]
*L. johnsonii* *	+	+					[181]
*L. reuteri*		+					[182]
*L. delbrueckii* subsp. *bulgaricus*, *Streptococcus thermophilus*	+						[183]
*L. acidophilus*, *L. rhamnosus*, *B. bifidum*, *B. lactis*	+	+					[184]
*L. acidophilus*, *L. rhamnosus*, *L. plantarum*, *L. delbrueckii* subsp. *bulgaricus*, *B. bifidum*	+	+					[185]
*L. acidophilus*, *L. rhamnosus*, *L. paracasei*, *B. lactis*, *B. breve*, *Pediococcus pentosaceus*		+					[172]
*L. acidophilus*, *L. plantarum*, *L. paracasei*, *L. delbrueckii* subsp. *bulgaricus*, *B. breve*, *B. longum, B. infantis*				±			[171]
*Lactobacillus* spp., *Bifidobacterium* spp., *Lactococcus* spp., *Propionibacterium* spp., *Acetobacter* spp.	+	+		±		+	[173]
*L. acidophilus*, *L. plantarum*, *L. delbrueckii* subsp. *bulgaricus*, *L. casei*, *B. breve*, *B. longum*, *B. infantis*, *S. thermophilus*		+					[186]
*Clostridium butyricum* *				+	+		[177]
*Saccharomyces boulardii* *	+	+					[176]

* Preclinical data given. +, definite positive effect; ±, possible positive effect; HCB, hepatocellular ballooning.

**Table 2:** Drug candidates and experimental agents with evidence of hepatoprotective activity in non-alcoholic fatty liver disease.

Name	Mechanism of Action	Development Phase	Liver-Related Effects						
			Cytolysis	Steatosis	HCB	Inflammation	Fibrosis	Cholestasis	References
Resmetirom	THRβ agonist	3	+	+	±	±	+	+	[11]
VK2809	THRβ agonist	2		+					[12]
ASC41 *	THRβ agonist	2		+	+	+	+		[13]
TERN-501 *	THRβ agonist	1	+	+			+		[15]
Firsocostat **	ACC inhibitor	2	+	+	+	+	+	±	[23]
Clesacostat **	ACC inhibitor	2		+					[24,31]
ASC40	FASN inhibitor	2	+	+		+	+		[16]
Aramchol	SCD1 inhibitor	3	+	+	+	+	+		[30]
Ervogastat	DGAT2 inhibitor	2		+					[31]
ION224	DGAT2 inhibitor	1		+			±		[216]
Docosahexaenoic acid	ω-3 PUFA	-		+					[40]
Epeleuton	ω-3 PUFA	2				±			[44]
Icosabutate	ω-3 PUFA	2	+			+	±	+	[46]
Eicosapentanoic acid *	ω-3 PUFA	-	+	+			+		[218]
Obeticholic acid	FXR agonist	3	+	+	+	+	+	+	[49,50]
EDP-305	FXR agonist	2	+	+			+	+	[54,56]
Tropifexor	FXR agonist	2	+	+			+	+	[63]
Cilofexor	FXR agonist	2		+			+	+	[64]
Vonafexor	FXR agonist	2	+	+				+	[65]
MET409	FXR agonist	2	+	+					[59]
TERN-101	FXR agonist	2	+	+					[67]
ASC42 *	FXR agonist	2		+		+	+		[16]
INT-767 *	FXR agonist	2		+	+	+	+		[58]
EDP-297 *	FXR agonist	1	+	+			+	±	[219]
BAR502 *	FXR agonist	-		+		+	+		[57]
GW4064 *	FXR agonist	-		+		+			[220]
Aldafermin	FGF19 analogue	2	+	+		+			[70]
Efruxifermin	FGF21 analogue	2	+	+	±	+	+		[71]
BIO89-100	FGF21 analogue	2	+	+					[72]
BFKB8488A	FGFR1c/KLB agonist	2		+					[74]
MK-3655	FGFR1c/KLB agonist	2		+					[221]
GLP-1-Fc-FGF21 D1 *	FGF21 analogue, GLP1R agonist	-	+	+					[75]
GB1211 *	galectin-3 antagonist	2					+		[88]
GM-CT-01 *	galectin-3/1 antagonist	-		+	+	+	+		[79]
JKB-122	TLR4 antagonist	2	+	+					[83]
Eritoran *	TLR4 antagonist	-	+			+	+		[81]
PXS-5153A *	LOXL2/3 inhibitor	-	+				+		[87]
Bezafibrate *	PPARα agonist	-		+	+	±			[98]
Pemafibrate	PPARα agonist	-	+				±	+	[97]
Fenofibrate	PPARα agonist	-	+	±	+				[93,94]
Gemfibrozil	PPARα agonist		+						[95]
Nifedipine *	PPARγ agonist	-	+				+		[101]
Seladelpar	PPARδ agonist	2	+					+	[103]
Saroglitazar	PPARα/γ agonist	2	+	+	+	+			[104,105]
Lanifibranor	PPARα/γ/δ agonist	3	+		+	+	+	+	[106]
Pioglitazone	PPARγ agonist, MPC inhibitor	-	+	+	+	+			[222]
Lobeglitazone	PPARγ agonist, MPC inhibitor	-	+	+					[100]
Azemiglitazone	MPC inhibitor	3	+	±	±	±			[107]
PXL065 *	MPC inhibitor	2		+		+	+		[109]
Semaglutide	GLP1R agonist	3		+		+			[113]
Exenatide	GLP1R agonist	-	+	+				+	[115,116]
Lixisenatide	GLP1R agonist	-		+		+	+		[119]
Liraglutide	GLP1R agonist	-		+	+	+	+	+	[120]
Dulaglutide	GLP1R agonist	-	+	+				+	[117,118]
Teduglutide *	GLP2R agonist	-		+	±	±			[113]
Tirzepatide	GLP1R/GIPR agonist	2	+	+					[123,124]
Cotadutide	GLP1R/GCGR agonist	2	+	+			±		[127]
Efinopegdutide *	GLP1R/GCGR agonist	2		+	±	±			[128]
Pemvidutide	GLP1R/GCGR agonist	1							[223]
HM15211 *	GLP1R/GCGR/GIPR agonist	2				+	+		[131]
Sitagliptin	DPP4 inhibitor	-		+	+	±			[134]
Vildagliptin	DPP4 inhibitor	-	+	+			+		[135]
Saxagliptin	DPP4 inhibitor	-	+	+		±		+	[136]
Alogliptin	DPP4 inhibitor	-				+			[139]
Omarigliptin	DPP4 inhibitor	-	+	+		+	+	+	[137]
Teneligliptin	DPP4 inhibitor	-	+	+					[138]
Evogliptin *	DPP4 inhibitor	-		+		+	+		[141]
Anagliptin *	DPP4 inhibitor	-			+	+	+		[142,143]
Trelagliptin *	DPP4 inhibitor	-	+	+		+		±	[144]
Gemigliptin *	DPP4 inhibitor	-		+		+	+		[145]
Linagliptin *	DPP4 inhibitor	-		+		+			[146]
Dapagliflozin	SGLT2 inhibitor	-	+	+			±	+	[150,224]
Empagliflozin	SGLT2 inhibitor	-	+	+	+		+	+	[151,225]
Canagliflozin	SGLT2 inhibitor	-	+	+			+	+	[152]
Ipragliflozin	SGLT2 inhibitor	-	±	+			+		[154,226]
Ertugliflozin	SGLT2 inhibitor	-	+						[156]
Remogliflozin	SGLT2 inhibitor	-	+				+		[155]
Luseogliflozin	SGLT2 inhibitor	-	+	+				+	[157,158]
Tofogliflozin	SGLT2 inhibitor	-	+	+			+	+	[159]
Licogliflozin **	SGLT1/2 inhibitor	2	+	+				+	[160,161]
SGL5213 *	iSGLT1 inhibitor	-		+	+	±	+		[162]
Miglitol	α-glucosidase inhibitor	-	+	+	±	+		+	[165]
Acarbose	α-glucosidase inhibitor	-	+						[164]
Voglibose *	α-glucosidase inhibitor	-		+					[166]
Liver-derived MSC	MSC	2				+	+		[192]
Umbilical cord-derived MSC *	MSC	-		+					[188]
Compact bone-derived MSC *	MSC	-		+	+	+	+		[189]
Tesamorelin	GHRH analogue	3	+	+					[196]
Berberine ursodeoxycholate	multimodal metabolic	2/1	+	+				+	[202]
Miricorilant	GR agonist/antagonist, MR antagonist	2		+					[203]
Nitazoxanide *	AMPK activator	2		+			+		[204,205]
PXL770	AMPK activator	2		+					[227]
Leucine + metformin + sildenafil	AMPK activator, eNOS activator	2		+					[228]
Pirfenidone *	multimodal antifibrotic	-	+				+		[209]
PBI-4547 *	GPCR84 antagonist	-		+	+	±			[229]
CpdA *	GPCR84 antagonist	-		+	+	+	+		[230]
CpdB *	GPCR84 antagonist	-		+	+	+	+		[230]
GPR120 agonist III *	GPCR120 agonist	-		+	±	+			[231]
Metabolitin *	GPRC6A agonist	-	+	+				+	[232]
SCO-267 *	GPR40 agonist	-	+	+			+		[233]
Evolocumab	PCSK9 inhibitor	-	±						[234]
Alirocumab	PCSK9 inhibitor	-	±						[234]
X203 *	IL11 antagonist	-	+	+		+	+		[212]
X209 *	IL11RA antagonist	-							[212]
Ezetimibe	NPC1L1 inhibitor	-	+	±	+				[235]
ORMD-0801	oral insulin	2		+					[236]
GalNAc-Stk25 ASO *	anti-STK25 ASO	-	+	+		+	+		[237]
Tipelukast *	LTR antagonist, PDE3/4 inhibitor, 5-LO/LT inhibitor	2		±	+	+	+		[210]
CM-101 *	CCL24 antagonist	2	+	±	±	±	+	±	[238]
Namodenoson	A3AR agonist	2	+						[214,239]
PLN-1474 *	α_v_β_1_ antagonist	1					+		[240]
CB4211	MOTS-c analogue	1	+	+					[241]
CER-209 *	P2Y13R agonist	1	+	+					[242]
DUR-928	multitarget epigenetic regulator	2	+	+			+	+	[217]
CRV431	cyclophilin A/B/D inhibitor	2	+						[243]
LPCN 1144	androgen receptor agonist	2		+	+	+	+		[244]
Osteocalcin *	N/A	-	+	+	+		+		[245]

* Preclinical data given; ** developed in combination; -, not in development. +, definite positive effect; ±, possible positive effect. HCB, hepatocellular ballooning; THRβ, thyroid hormone receptor β; ACC, acetyl-CoA carboxylase; FASN, fatty acid synthase; SCD1, stearoyl-CoA desaturase 1; DGAT2, diglyceride acyltransferase 2; PUFA, polyunsaturated fatty acid; FXR, farnesoid X receptor; FGF, fibroblast growth factor; FGFR1c/KLB, FGF receptor/β-klotho complex; TLR4, toll-like receptor 4; LOXL, lysyl oxidase-like protein; PPAR, peroxisome proliferator-activated receptor; MPC, mitochondrial pyruvate carrier; GLP1R, glucagon-like peptide 1 receptor; GIPR, glucose-dependent insulinotropic polypeptide receptor; GCGR, glucagon receptor; DPP4, dipeptidyl peptidase 4; SGLT, sodium/glucose cotransporter; iSGLT1, intestinal SGLT 1; MSC, mesenchymal stromal cells; GHRH, growth hormone releasing hormone; GR, glucocorticoid receptor; MR, mineralocorticoid receptor; AMPK, adenosine monophosphate-activated protein kinase; eNOS, endothelial nitric oxide synthase; GPCR, G protein-coupled receptor; GPRC6A, G protein-coupled receptor family C group 6 member A; PCSK9, proprotein convertase subtilisin/kexin type 9; IL11, interleukin 11; IL11RA, IL11 receptor α subunit; NPC1L1, Niemann-Pick C1-like protein 1; STK25, serine/threonine kinase 25; ASO, antisense nucleotide; LTR, leukotriene receptor; PDE, phosphodiesterase; 5-LO/LT, 5-lipoxygenase/leukotriene pathway; CCL24, C-C motif chemokine ligand 24; A3AR, A3 adenosine receptor; MOTS-c, mitochondrial open reading frame of the twelve S ribosomal ribonucleic acid-c; P2Y13R, P2Y purinergic receptor 13.

## Data Availability

Part of the data analyzed in this study are openly available at ClinicalTrials.gov, https://www.clinicaltrials.gov/ct2/home (accessed on 10 December 2021).
